# Predictive factors of the treatment outcome in patients with advanced biliary tract cancer receiving gemcitabine plus cisplatin as first-line chemotherapy

**DOI:** 10.1007/s00535-018-1518-3

**Published:** 2018-10-08

**Authors:** Yuko Suzuki, Motoyasu Kan, Gen Kimura, Kumiko Umemoto, Kazuo Watanabe, Mitsuhito Sasaki, Hideaki Takahashi, Yusuke Hashimoto, Hiroshi Imaoka, Izumi Ohno, Shuichi Mitsunaga, Masafumi Ikeda

**Affiliations:** Department of Hepatobiliary and Pancreatic Oncology, National Cancer Center Hospital East, 6-5-1 Kashiwanoha, Kashiwa, 277-8577 Japan

**Keywords:** Biliary tract cancer, Prognostic factor, Validation, Chemotherapy, Gemcitabine and cisplatin

## Abstract

**Background:**

Few studies have clearly identified the prognostic factors in patients with advanced biliary tract cancer (BTC) receiving gemcitabine plus cisplatin (GC) which is acknowledged as standard chemotherapy regimen.

**Objectives:**

The aim of this study was to identify predictive factors of the overall survival (OS) in advanced BTC patients receiving GC therapy.

**Methods:**

Data of 307 patients with advanced BTC who received GC therapy as the first-line chemotherapy at our institution from January 2007 to June 2017 were reviewed retrospectively. The patients were randomly assigned to the investigation or the validation dataset at the ratio of 2:1. Multivariate analysis was conducted to identify the prognostic factors, a prognostic index is proposed from the investigation dataset, and the usefulness of this index was confirmed in the validation dataset.

**Results:**

Multivariate analysis identified poor performance status, elevated serum lactate dehydrogenase, and elevated neutrophil-to-lymphocyte ratio as independent unfavorable predictors. The patients could be classified into three groups according to these factors, and it was found that the outcomes differed significantly among the three groups (*P* = 0.0002, good- vs. intermediate-prognosis groups; *P* = 0.005, intermediate- vs. poor-prognosis groups). When this index was applied to the validation dataset, the OS was confirmed to differ significantly among the three groups (*P* = 0.04, good- vs. intermediate-prognosis groups, *P* < 0.0001, intermediate- vs. poor-prognosis groups).

**Conclusions:**

We identified three predictors of the OS in patients with advanced BTC receiving GC therapy in this study, based on which we could classify the patients into three risk groups.

## Introduction

Biliary tract cancer (BTC), while being an uncommon malignancy in Western countries, is relatively common, accounting for 2–3% of all malignant neoplasms, in Japan; approximately 22,000 new patients are registered and 18,000 die of the disease each year in Japan [1]. BTC has a dismal prognosis and surgical resection is the only treatment modality that offers any chance of cure. However, in many patients, the diagnosis is made only at an advanced stage of the disease, by which time, surgical resection is no longer applicable. On the other hand, even in patients undergoing curative resection, recurrence occurs at a very high rate [2–4]. Systemic chemotherapy plays an important role in the treatment of unresectable or recurrent BTC. In a phase III trial (ABC-02) conducted in the United Kingdom (UK), gemcitabine plus cisplatin (GC) therapy improved the survival outcome (median overall survival [OS], 11.7 months) as compared to treatment with gemcitabine alone [median OS, 8.1 months, hazard ratio, 0.64; 95% confidence interval (CI) 0.52–0.80; *P* < 0.0001] [5]. In a randomized phase II trial (the BT22 trial) conducted in Japan at around the same time, a similar efficacy and safety of GC therapy were observed (median OS: GC, 11.2 months; gemcitabine alone, 7.7 months; hazard ratio, 0.69; 95% CI 0.42–0.80; *P* < 0.0001) [6]. Based on the above-described results, GC is now established as the standard first-line chemotherapy for advanced BTC.

Subsequently, a randomized phase III trial (JCOG1113) conducted in Japan showed the non-inferiority of the combined gemcitabine plus S-1 (GS) therapy to GC therapy in terms of the overall survival outcome [7]. Therefore, GS is also currently available as one of the chemotherapy options for patients with advanced BTC, although GC still remains the standard first-line regimen. It is important to precisely identify patients who can derive survival benefit from GC therapy. However, from the few studies conducted until date, no predictors of the OS have been identified yet. In addition, few prognostic indexes have been constructed and few validation studies of the prognostic index have been conducted [8–12]. The purpose of this study, therefore, was to identify and then conduct the prognostic index of OS, and validate it, in patients with advanced BTC receiving GC as first-line chemotherapy.

## Materials and methods

### Patients

This study included a total of 307 patients with histologically or cytologically proven advanced BTC, extrahepatic cholangiocarcinoma, intrahepatic cholangiocarcinoma, gallbladder or ampullary carcinoma, who were started on GC as first-line chemotherapy at the National Cancer Center Hospital East, Kashiwa, Japan, between January 2007 and June 2017. All clinical data were reviewed retrospectively from the hospital records. The database was fixed for analysis in December 2017. All patients were randomly assigned to the investigation dataset (205 patients) or the validation dataset (102 patients) at the ratio of 2:1. This retrospective study was conducted in accordance with the 1964 Declaration of Helsinki and its later amendments, and the protocol was approved by the Ethics Committee of National Cancer Center Hospital East (Approval no. 2017-322).

### Treatment

All patients received GC therapy: cisplatin (25 mg/m^2^), followed by gemcitabine (1000 mg/m^2^) administered by intravenous infusion on days 1 and 8 of each 3-week cycle. A total of 16 doses of cisplatin was administered (400 mg/m^2^), unless there was evidence of disease progression or unacceptable toxicity, while gemcitabine alone was continued indefinitely until evidence of disease progression or appearance of unacceptable toxicity.

### Assessment of toxicity and efficacy

All patients underwent physical examination and assessments for evidence of drug toxicity before and every 1 or 2 weeks after the initiation of GC therapy. Toxicities appearing during GC therapy were graded according to the Common Terminology Criteria for Adverse Events (CTCAE) version 4.0. Computed tomography (CT) or magnetic resonance imaging (MRI) was performed every 4–8 weeks, the tumor responses were assessed on the images by both medical oncologists and radiologists, in accordance with the Response Evaluation Criteria In Solid Tumors (RECIST) version 1.1, and the best response in each patient was recorded.

### Statistical analysis

OS was calculated as the time interval from the date of initiation of the GC therapy until the date of death. Progression-free survival (PFS) was calculated from the date of initiation of the GC therapy until the date of documentation of disease progression or death. Patients who did not show disease progression and patients who died were excluded at the date of their last follow-up visit or the date of their death. Univariate analysis was performed using Mann–Whitney *U* test for continuous variables and Chi-squared test for categorical variables. The Kaplan–Meier method was used to estimate the time-to-event distribution, and *P* values were calculated using a log-rank test. Hazard ratios were calculated using Cox proportional hazard model. Age, body mass index, maximum tumor size and laboratory parameters were set as continuous variables, while other factors were used as categorical variables. Statistically significant variables (*P* < 0.05) identified by univariate analysis were entered into the multivariate analysis model. After identification of the prognostic factors by multivariate analysis, the continuous variables were also divided into two categories, followed by receiver operating characteristics (ROC) curve analysis to construct a prognostic index. ROC curve was used to determine the optimal cutoff value that predicted the survival and maximized both the sensitivity and the specificity of continuous variables. The prognostic index was calculated based on the statistically significant prognostic factors identified by multivariate analysis. All tests were two sided and *P* < 0.05 was considered as denoting statistical significance. All the statistical analyses were performed using the JMP 13.0 software for Macintosh, version 13.2 (SAS Institute Inc., Cary, North Carolina, USA).

## Results

### Patient characteristics

A total of 307 patients with advanced BTC received GC as the first-line chemotherapy at our institution between January 2007 and June 2017. By the time of the analysis, 226 had already died. The baseline characteristics and clinical data of all the patients (*n* = 307) included in the investigation dataset (*n* = 205) and validation dataset (*n* = 102) are summarized in Table 1. Although there were a few missing data on the patient characteristics (serum CEA level data missing in 2 patients, and serum CA19-9 level data missing in 3 patients), there were no significant differences in the patient characteristics between the investigation and validation datasets.

**Table 1:** Baseline patient characteristics

Variable	Total (*n* = 307)	Investigation dataset (*n* = 205)	Validation dataset (*n* = 102)	*P* value*
Age, years				
Median [range]	68 [33–85]	68 [35–83]	68 [33–85]	0.81
Gender, *n* (%)				
Male	179 (58.3)	116 (56.6)	63 (61.8)	0.38
Female	128 (41.7)	89 (43.4)	39 (38.2)	
ECOG PS, n (%)				
0	199 (64.8)	130 (63.4)	69 (67.7)	0.46
1–2	108 (35.2)	75 (36.6)	33 (32.3)	
Primary tumor site, *n* (%)				
Intrahepatic bile duct	103 (33.6)	66 (32.2)	37 (36.3)	0.53
Extrahepatic bile duct	84 (27.3)	55 (26.8)	29 (28.4)	
Gallbladder	108 (35.2)	74 (36.1)	34 (33.3)	
Ampulla of Vater	12 (3.9)	10 (4.9)	2 (2.0)	
Maximum tumor size, mm, median [range]	40 [5–165]	39 [9–165]	36 [5–140]	0.54
Extent of disease, *n* (%)				
Locally advanced	62 (20.2)	41 (20.0)	21 (20.6)	0.90
Metastatic	245 (79.8)	164 (80.0)	81 (79.4)	
Metastatic site, *n* (%)				
Liver	80 (26.1)	55 (26.8)	25 (24.5)	0.66
Lung	35 (11.4)	25 (12.2)	10 (9.8)	0.53
Lymph node	179 (58.3)	122 (59.5)	57 (55.9)	0.54
Peritoneum	85 (27.8)	52 (25.4)	33 (32.4)	0.20
Type of tumor, *n* (%)				
Adenocarcinoma	293 (95.4)	194 (94.6)	99 (97.1)	0.44
Adenosquamous carcinoma	3 (1.0)	2 (1.0)	1 (1.0)	
Others^a^	11 (3.6)	9 (4.4)	2 (1.9)	
Prior surgical resection, *n* (%)	72 (23.5)	48 (23.4)	24 (23.5)	0.98
Biliary drainage, *n* (%)	135 (43.9)	90 (43.9)	45 (44.1)	0.97
Subsequent chemotherapy, *n* (%)	181 (58.9)	120 (58.5)	61 (59.8)	0.83
Blood examinations, median [range]				
White blood cell count/μL	6000 [2100–26,100]	5900 [2100–26,100]	6100 [2500–25,900]	0.94
Hemoglobin, g/dL	12.0 [7.3–19.0]	12.0 [7.3–19.0]	12.0 [7.9–15.1]	0.93
Platelets,/μL	20.9 [7.9–67.4]	20.5 [8.0–44.3]	21.8 [7.9–67.4]	0.44
Albumin, g/dL	3.8 [2.0–5.2]	3.8 [2.2–5.2]	3.7 [2.0–4.7]	0.42
Total bilirubin, mg/dL	0.7 [0.2–3.0]	0.7 [0.2–3.0]	0.8 [0.3–2.8]	0.96
ALP, IU/L	443 [75–3395]	429 [158–3395]	501 [75–3056]	0.24
LDH, IU/L	180 [85–1211]	180 [85–1211]	179 [111–973]	0.94
CRP, mg/dL	0.65 [0.03–18.35]			
NLR	2.99 [0.51–25.9]	2.91 [0.51–17.4]	3.11 [1.06–25.9]	0.77
Tumor marker, median [range]				
CEA, ng/mL	3.8 [0.2–1482]	3.8 [0.2–1482]	4.0 [0.3–606]	0.57
CA19-9, U/mL	168 [0.1–129,820]	155 [0.1–129,820]	209 [0.1–68,120]	0.78

*ECOG PS* Eastern Cooperative Oncology Group Performance Status, *ALP* alkaline phosphatase, *LDH* lactate dehydrogenase, *CRP* C-reactive protein, *NLR* neutrophil-to-lymphocyte ratio, *CEA* carcinoembryonic antigen, *CA19*-*9* carbohydrate antigen 19-9

*The difference between the investigation dataset and validation dataset

^a^These patients were diagnosed by cytology class V with imaging findings

### Efficacy of GC

**Fig. 1:**
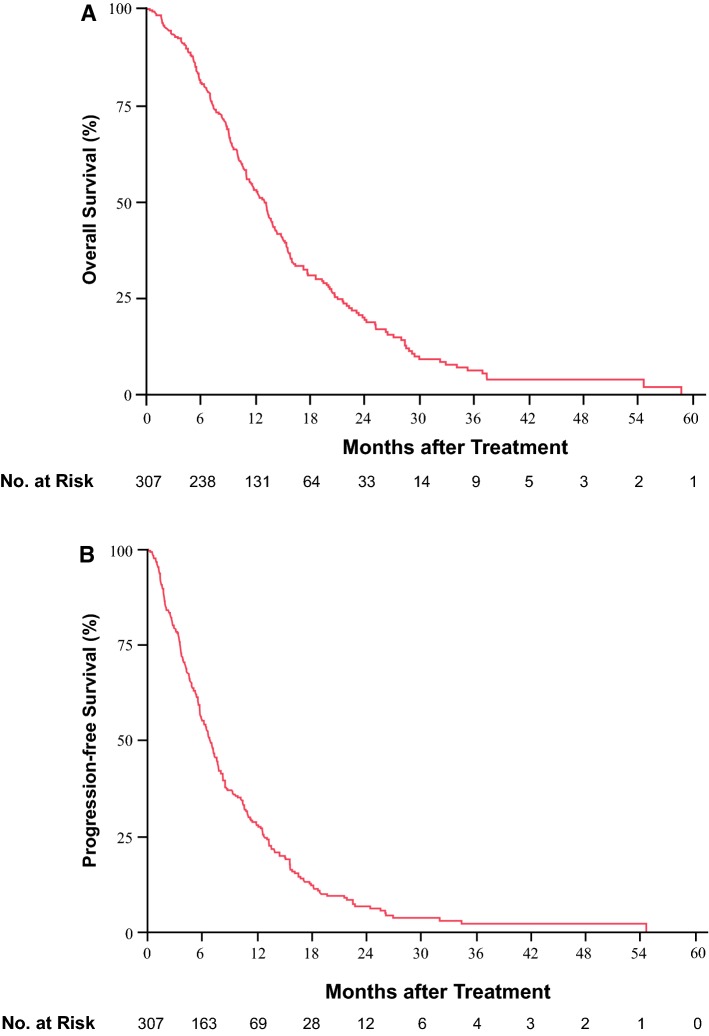
Kaplan–Meier curves of OS (**a**) and PFS (**b**) in patients with advanced BTC receiving gemcitabine plus cisplatin (GC) therapy. The median OS and PFS were 13.0 months (95% CI 11.0–13.9) and 6.9 months (95% CI 5.9–7.7), respectively. *OS* overall survival, *PFS* progression-free survival*, CI* confidence interval

The objective tumor response could be assessed by CT/MRI in a total of 304 patients, in accordance with RECIST version 1.1 (Table 2). Complete response (CR), partial response (PR), and stable disease (SD) were observed in 9 (2.9%), 43 (14.0%), and 196 (63.9%) patients, respectively, representing an overall response rate (CR + PR) of 16.9% and tumor control rate (CR + PR + SD) of 80.8%. Moreover, two patients each who showed CR and PR underwent curative resection after the GC therapy. Figure 1 shows the Kaplan–Meier curves for OS (Fig. 1a) and PFS (Fig. 1b) in the entire study population of 307 patients that had received GC therapy. The median OS, PFS and 1-year survival rate were 13.0 months [95% confidence interval (CI) 11.0–13.9], 6.9 months (95% CI 5.9–7.7), and 52.7%, respectively.

**Table 2:** Best tumor response according to RECIST version 1.0

	Total, *n* (%)	Investigation dataset (*n* = 205)	Validation dataset (*n* = 102)	*P* value*
Best response				
CR	9 (2.9)	8 (3.9)	1 (1.0)	0.51
PR	43 (14.0)	30 (14.6)	13 (12.8)	
SD	196 (63.9)	126 (61.5)	70 (68.6)	
PD	56 (18.2)	39 (19.0)	17 (16.7)	
NE	3 (1.0)	2 (1.0)	1 (1.0)	
ORR (CR + PR)	52 (16.9)	38 (18.5)	14 (13.7)	0.29
DCR (CR + PR + SD)	248 (80.8)	164 (80.0)	84 (82.4)	0.62

*CR* complete response, *PR* partial response, *SD* stable disease, *PD* progressive disease, *NE* not evaluable, *ORR* overall response rate, *DCR* disease control rate

*The difference between the investigation dataset and validation dataset

There were no significant differences in the efficacy of GC therapy between the investigation and validation datasets (Table 2).

### Treatment and toxicity

The median duration of treatment was 6.0 months (range 0.03–47.7 months). The GC therapy had to be discontinued in 292 patients (95.1%), the main reasons for the treatment discontinuation being disease progression (247 patients, 80.5%) and treatment-related toxicities (17 patients, 5.5%). A total of 181 patients (58.9%) received subsequent therapies after failure of GC therapy. In most of these cases (*n* = 152, 84.0%), the second-line treatment was S-1 monotherapy. The toxicity of GC therapy is summarized in Table 3. The mainly encountered grade 3 or more severe toxicities were neutropenia (172 patients, 56.0%), anemia (102 patients, 33.2%), leukopenia (101 patients, 32.9%), lymphocytes decreased (70 patients, 22.8%), thrombocytopenia (41 patients, 13.3%), alkaline phosphatase elevated (38 patients, 12.4%), and aspartate aminotransferase elevated (37 patients, 12.1%); there were no treatment-related deaths. Dose modifications of gemcitabine and cisplatin were needed in 40.7% and 29.3% of all patients, respectively.

**Table 3:** Treatment-related toxicity graded according to CTCAE version 4.0

Variable	Toxicity grade, *n* (%)		
	Grade 3	Grade 4	Any grades
Hematological			
Leucopenia	97 (31.6)	4 (1.3)	283 (92.2)
Anemia	93 (30.3)	9 (2.9)	304 (99.0)
Thrombocytopenia	28 (9.1)	13 (4.2)	223 (72.6)
Neutropenia	127 (41.4)	45 (14.7)	267 (87.0)
Lymphocyte decreased	63 (20.5)	7 (2.3)	223 (72.6)
Non-hematological			
Fatigue	3 (1.0)	0 (0)	172 (56.0)
Anorexia	5 (1.6)	0 (0)	141 (45.9)
Nausea	1 (0.3)	0 (0)	101 (32.9)
Vomiting	1 (0.3)	0 (0)	16 (5.2)
Febrile neutropenia	5 (1.6)	0 (0)	5 (1.6)
Alopecia	0 (0)	0 (0)	29 (9.4)
Neuropathy	2 (0.7)	0 (0)	42 (13.4)
Thrombosis	4 (1.3)	2 (0.7)	11 (3.6)
AST elevated	37 (12.1)	0 (0)	213 (69.4)
ALT elevated	34 (11.1)	0 (0)	201 (65.5)
ALP elevated	38 (12.4)	0 (0)	258 (84.0)
Creatinine increased	5 (1.6)	0 (0)	137 (44.6)
Albumin decreased	15 (4.9)	0 (0)	265 (86.3)
Hyponatremia	26 (8.5)	0 (0)	188 (61.2)

*AST* aspartate aminotransferase, *ALT* alanine aminotransferase, *ALP* alkaline phosphatase

### Identification of predictive factors of the survival outcome and construction of a prognostic index from the investigation dataset

The results of univariate analysis of a total of 22 variables in the investigation dataset are shown in Table 4. Among these variables, a total of 13 factors recorded before the start of GC therapy were associated with a worse OS. Multivariate analysis identified poor performance status (PS), increased serum lactate dehydrogenase (LDH) level, and elevated neutrophil-to-lymphocyte ratio (NLR) as independent predictors of a worse OS (Table 4).

**Table 4:** Univariate and multivariate analyses using a Cox proportional hazard model to identify predictors of the overall survival

Variable	Univariate analysis				Multivariate analysis		
	OS, months^a^	HR	95% CI	*P* value	HR	95% CI	*P* value
Age		1.010	0.991–1.031	0.30			
Gender							
Male		1.000					
Female	12.4	1.110	0.799–1.536	0.53			
Body Mass Index	13.2	0.992	0.936–1.050	0.78			
ECOG PS							
0	13.9	1.000			1.000		
1–2	7.6	2.383	1.699–3.327	< 0.001	1.709	1.162–2.497	< 0.001
Primary tumor site							
Bile duct^b^	13.2	1.000					
Gallbladder	11.3	1.235	0.877–1.721	0.22			
Maximum tumor size		1.007	1.003–1.012	0.002	0.995	0.988–1.0008	0.092
Extent of disease							
Locally advanced	18.6	1.000			1.000		
Metastatic	11.6	1.509	1.023–2.298	0.038	1.579	1.163–2.493	0.067
Liver metastasis							
Absent	13.3	1.000					
Present	10.2	1.424	0.979–2.034	0.064			
Peritoneal dissemination							
Absent	13.6	1.000			1.000		
Present	8.8	1.559	1.074–2.219	0.020	1.218	0.805–1.816	0.35
Prior surgical resection							
Present	16.4	1.000			1.000		
Absent	11.6	1.718	1.151–2.662	0.007	1.580	0.970–2.618	0.19
Biliary drainage							
Present	12.2	1.000					
Absent	13.2	1.044	0.756–1.450	0.79			
White blood cells count		1.0001	1.00004–1.0001	0.001	0.999	0.999–1.0001	0.82
Hemoglobin		0.844	0.766–0.929	< 0.001	1.037	0.908–1.185	0.59
Platelets		0.990	0.970–1.009	0.33			
Albumin		0.510	0.388–0.673	< 0.001	0.642	0.401–1.020	0.061
Total bilirubin		1.210	0.889–1.615	0.22			
LDH		1.003	1.002–1.004	< 0.001	1.002	1.0005–1.003	0.006
ALP		1.0003	1.00004–1.00006	0.027	1.0003	0.999–1.0006	0.11
CRP		1.120	1.065–1.170	< 0.001	1.009	0.923–1.092	0.84
NLR		1.201	1.125–1.278	< 0.001	1.117	1.019–1.219	0.018
CEA		1.001	0.999–1.002	0.13			
CA19-9		1.00002	1.00001–1.00002	0.007	1.00001	0.999–1.00002	0.19

*OS* overall survival, *HR* hazard ratio, *CI* confidence interval, *LDH* lactate dehydrogenase, *ALP* alkaline phosphatase, CRP C-reactive protein, neutrophil-to-lymphocyte ratio, *CEA* carcinoembryonic antigen, *CA19*-*9* carbohydrate antigen 19-9, *HR* hazard ratio, *CI* confidence interval

^a^OS was shown for only categorical variables

^b^Bile duct; intrahepatic bile duct, extrahepatic bile duct and ampulla of Vater

**Fig. 2:**
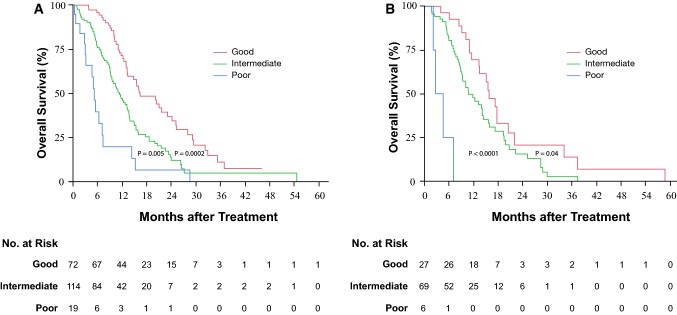
Kaplan–Meier curves of overall survival according to the risk groups based on the number of poor prognostic factors in the investigation dataset (**a**) and validation dataset (**b**). The patients were divided into three risk groups as follows: (1) good-prognosis group, none of the poor prognostic factors present; (2) intermediate-prognosis group, 1 or 2 poor prognostic factors present; (3) poor-prognosis group, all poor prognostic factors present. The three poor prognostic factors were elevated serum lactate dehydrogenase (cutoff value, 245 IU/L; upper limit of normal), elevated neutrophil-to-lymphocyte ratio (cutoff value, 3.0), and poor performance status (ECOG PS 0 vs. 1 or 2). There were statistically significant differences in the overall survival among the three groups in both the investigation and validation datasets. *ECOG PS* Eastern Cooperative Oncology Group Performance Status

For clinical application, we constructed a prognostic index based on the three prognostic factors identified by multivariate analysis, namely PS, serum LDH level and the NLR. Then, on the basis of this index, the patients could be classified into three risk groups. Because the hazard ratios calculated by multivariate analysis after categorizing the serum LDH and NLR into two variables (data not shown) did not differ among the three identified prognostic factors, we calculated the prognostic index on the basis of the number of independent prognostic factors. The cutoff serum LDH level was determined as upper limit of normal (245 IU/L) because the cutoff value calculated from the ROC curve was approximately equivalent to the upper limit of normal. The cutoff value for the NLR was determined to be 3.0, because the cutoff value calculated from the ROC curve was almost equivalent to that determined in previous investigations [8, 13–15]. Then the patients were divided into three groups, as follows: the good-prognosis group [none of the poor prognostic factors present, 72 patients (35.1%)], intermediate- prognosis group [1 or 2 poor prognostic factors present, 114 patients (55.6%)], and poor-prognosis group [all three poor prognostic factors present, 19 patients (9.3%)]. The median OS times in the good-, intermediate-, and poor-prognosis groups were 16.3 months (95% CI 13.2–23.1), 11.3 months (95% CI 9.2–13.4), and 5.3 months (95% CI 3.0–7.1), respectively. Thus, the outcomes were found to differ significantly among the three groups: good- vs. intermediate-prognosis groups, *P* = 0.0002 and intermediate- vs. poor-prognosis groups, *P* = 0.005 (Fig. 2a).

### Validation of the prognostic index in the validation set

The median OS, PFS and 1-year survival rate in the validation dataset were 13.2 months (95% CI 10.0–15.5), 5.9 months (95% CI 4.5–7.2), and 51.8%, respectively. The prognostic index constructed from the investigation dataset was applied to the validation dataset. In the validation dataset, the median OS in the good-, intermediate-, and poor-prognosis groups were 15.8 months (95% CI 11.5–20.5), 10.8 months (95% CI 8.9–14.4), and 3.6 months (95% CI 2.1–7.0), respectively (Fig. 2b). The differences among the three groups were statistically significant: good- vs. intermediate-prognosis group, *P* = 0.04, and intermediate- vs. poor-prognosis groups, *P* < 0.0001.

## Discussion

In patients with advanced BTC, systemic chemotherapy is one of the important treatment modalities to improve survival. While some investigators have reported on the prognostic factors in advanced BTC patients receiving GC therapy, these prognostic factors had not yet been confirmed as valid, because of the limited number of patients enrolled. The present study was aimed at evaluating the efficacy and safety of GC therapy, and identifying the predictive factors of the OS in a relatively large number of the patients, that is, over 300 patients, with advanced BTC receiving GC therapy. The efficacy and safety parameters of GC therapy at our institution were almost similar to those reported from the phase III trial of GC therapy (the ABC-02 trial) conducted in the UK. In our patient cohort, we identified three factors (poor PS, increased serum LDH level and elevated NLR) as independent predictors of an unfavorable OS from among 13 potential factors by multivariate analysis of the data from the 205 patients included in the investigation dataset. We constructed a prognostic index for clinical application based on these three independent prognostic factors, and then confirmed the usefulness of this prognostic index in the 102 patients of the validation dataset.

PS was identified as one of the most robust and important prognostic factors in patients with various advanced cancer. Although PS is a somewhat subjective and vague assessment of the physical condition of cancer patients, it was identified as the most important predictor of the survival in advanced BTC patients receiving chemotherapy. Indeed, some previous studies have also reported PS as a prognostic factor in patients with advanced BTC, in conformity with our findings [8–11, 13, 14, 16–21]. Although previous reports suggest a poor prognosis in patients with PS 2 [8, 10, 11, 17, 18, 21], both patients with PS 2 and PS 1 had a poor prognosis in this study. The median OS in the patients with PS 0, PS 1 and PS 2 in this study was 13.9, 9.0 and 5.4 months, respectively. While the difference in the OS between the PS 0 and PS 1 patients was significant (*P* < 0.0001), that between the patients with PS 1 and PS 2 was not significant (*P* = 0.16). Therefore, we divided the cohort into patients with PS 0 and PS 1–2 for predicting the prognosis. Our results were consistent with those described in some other reports [9, 16, 19, 20].

LDH is a glycolytic enzyme with a key role in the conversion of pyruvate to lactate under anaerobic conditions. In hypoxic environments, as in tumor tissues, hypoxia-inducible factor 1α (HIF-1α) is commonly induced, which activates both LDH-A, which is one of the LDH isozymes, and pro-angiogenesis factors such as vascular endothelial growth factor (VEGFA, VEGFR) via the same molecular pathway [22, 23]. Therefore, elevated serum LDH might be an indirect marker of more aggressive tumor angiogenesis and a higher tumor burden, and thereby of a poor prognosis [24–26]. Elevated serum LDH has been reported to be associated with chemo-resistance to several anticancer-drugs such as paclitaxel, cetuximab and gemcitabine [27–29]. Therefore, consistent with previous reports to the previous studies [12, 18, 19, 30], our study also suggested that elevated serum LDH might be associated with a worse OS in patients with advanced BTC receiving GC therapy.

Elevated NLR had been recognized as an indicator of a poor prognosis [31, 32] and poor tumor response [33, 34] in many cancers. Several investigators have reported the value of NLR as a predictor of the OS [8, 13, 14, 35, 36]. An elevated NLR might represent induced immunocompetence or neutrophilia. It is well known that neutrophilia contributes to stimulating the tumor microenvironment, specifically promoting cell proliferation, angiogenesis, invasion and metastasis in cancer [14, 35, 37, 38]. Neutrophilia also plays a role in inhibiting the immune system by suppressing the cytolytic activity of immune cells such as lymphocytes, T cells and natural killer cells [39]. On the other hand, lymphocytes are known as indispensable mediators in the anti-tumor immune system. Some previous studies have revealed that a decreased count of lymphocytes in a tumor is associated with a worse response to chemotherapy and a poor prognosis in cancer patients [32, 40–42]. Therefore, an elevated NLR might be associated with potential tumor growth.

Using the three aforementioned prognostic factors, we constructed a prognostic index, based on which we could divide our patients into three different prognosis groups, and the usefulness of this index was confirmed in the validation dataset. This index is simple and easy to apply for the prediction of the prognosis prior to the initiation of chemotherapy in the daily clinical setting. The OS in the poor-prognosis group, with all three poor prognostic factors, was extremely dismal (median OS, 3.6–5.3 months). Therefore, it would be desirable for such patients to be offered the best supportive care or a more conservative regimen. This index may be helpful in predicting the life expectancy in advanced BTC patients receiving GC therapy and be useful to stratify patients in future clinical trials.

In this study, nine patients showed CR, and five of these patients with CR were still alive at the time of the analysis. One of these patients, and also one patient who showed PR, underwent conversion surgery after the GC therapy. Although GC therapy generally serves as palliative chemotherapy in patients with advanced BTC, it may have a potential role in enabling conversion surgery.

There were three major limitations of this study. First, as this study was conducted retrospectively, we could not include any pre-treatment data, such as weight loss, intensity of pain, or quality of life, which were not fully documented in the hospital records. Second, external validation could not have performed to allow generalizability of our findings, because this was a single-institution study. Third, these predictive factors were not specific to advanced BTC. Although we included some specific factors in patients with advanced BTC in the analysis as potential predictive factors, such as the serum total bilirubin level, biliary drainage, tumor size, serum CA19-9 level and presence/absence of metastatic disease, none of these factors was identified as a predictor of the OS in our cohort. Further investigation to identify other novel biliary cancer-specific markers is needed. In contrast, the strength of this study was the large sample size with few missing patient data recruited from a major Japanese cancer center. In addition, the patient selections, management of GC therapy, and assessment of the tumor response were unified as this was a single-institution study. Therefore, because of the solid and similar patient data, the efficacy and safety of GC therapy in our cohort were comparable to that reported from the ABC-02 and BT-22 trials.

In conclusion, we identified three predictive factors of the OS in advanced BTC patients receiving GC therapy, which allowed these patients to be classified into three risk groups. These findings are expected to be helpful in decision-making on the first-line chemotherapy and survival estimation in patients with advanced BTC.
